# Differing Burden and Epidemiology of Non-Typhi *Salmonella* Bacteremia in Rural and Urban Kenya, 2006–2009

**DOI:** 10.1371/journal.pone.0031237

**Published:** 2012-02-21

**Authors:** Collins Tabu, Robert F. Breiman, Benjamin Ochieng, Barrack Aura, Leonard Cosmas, Allan Audi, Beatrice Olack, Godfrey Bigogo, Juliette R. Ongus, Patricia Fields, Eric Mintz, Deron Burton, Joe Oundo, Daniel R. Feikin

**Affiliations:** 1 Field Epidemiology and Laboratory Training Program, Nairobi, Kenya; 2 Ministry of Public Health and Sanitation, Nairobi, Kenya; 3 Kenya Medical Research Institute/Centers for Disease Control and Prevention, Nairobi, Kenya; 4 Jomo Kenyatta University of Agriculture and Technology, Nairobi, Kenya; 5 International Emerging Infections Program, Global Disease Detection, Centers for Disease Control and Prevention, Nairobi, Kenya; 6 Division of Foodborne, Waterborne, and Environmental Diseases, Centers For Disease Control and Prevention, Atlanta, Georgia, United States of America; Indian Institute of Science, India

## Abstract

**Background:**

The epidemiology of non-Typhi *Salmonella* (NTS) bacteremia in Africa will likely evolve as potential co-factors, such as HIV, malaria, and urbanization, also change.

**Methods:**

As part of population-based surveillance among 55,000 persons in malaria-endemic, rural and malaria-nonendemic, urban Kenya from 2006–2009, blood cultures were obtained from patients presenting to referral clinics with fever ≥38.0°C or severe acute respiratory infection. Incidence rates were adjusted based on persons with compatible illnesses, but whose blood was not cultured.

**Results:**

NTS accounted for 60/155 (39%) of blood culture isolates in the rural and 7/230 (3%) in the urban sites. The adjusted incidence in the rural site was 568/100,000 person-years, and the urban site was 51/100,000 person-years. In both sites, the incidence was highest in children <5 years old. The NTS-to-typhoid bacteremia ratio in the rural site was 4.6 and in the urban site was 0.05. *S.* Typhimurium represented >85% of blood NTS isolates in both sites, but only 21% (urban) and 64% (rural) of stool NTS isolates. Overall, 76% of *S.* Typhimurium blood isolates were multi-drug resistant, most of which had an identical profile in Pulse Field Gel Electrophoresis. In the rural site, the incidence of NTS bacteremia increased during the study period, concomitant with rising malaria prevalence (monthly correlation of malaria positive blood smears and NTS bacteremia cases, Spearman's correlation, p = 0.018 for children, p = 0.16 adults). In the rural site, 80% of adults with NTS bacteremia were HIV-infected. Six of 7 deaths within 90 days of NTS bacteremia had HIV/AIDS as the primary cause of death assigned on verbal autopsy.

**Conclusions:**

NTS caused the majority of bacteremias in rural Kenya, but typhoid predominated in urban Kenya, which most likely reflects differences in malaria endemicity. Control measures for malaria, as well as HIV, will likely decrease the burden of NTS bacteremia in Africa.

## Introduction

Non-Typhi *Salmonella* (NTS) is a common cause of bacteremia among children and adults in Africa [1]–[12]. NTS bacteremia in Africa is highly associated with other diseases, particularly HIV and malnutrition, and possibly malaria [2], [9], [10], [12]. As the epidemiology of these associated diseases evolves in Africa, the epidemiology and burden of NTS bacteremia may also change. Moreover, recent reports of a multi-drug resistant NTS clone, causing invasive disease in Africa suggests that the epidemiology of NTS bacteremia might also be in flux due to other selective pressures [13], [14]. Lastly, as more Africans migrate to urban areas, living in crowded, squalid conditions, these new environments might also lead to a different epidemiology of NTS bacteremia. Updated epidemiologic data on NTS bacteremia can serve to guide future policy decisions on treatment and prevention.

We evaluated three years of population-based surveillance on NTS bacteremia from an urban informal settlement in Nairobi and a rural area of western Kenya. Our surveillance highlights stark differences in invasive *Salmonella* epidemiology in urban and rural Africa and the close associations between NTS bacteremia and HIV and malaria.

## Methods

### Study sites

The Centers for Disease Control and Prevention's (CDC) International Emerging Infections Program (IEIP) and the Kenya Medical Research Institute (KEMRI) have conducted population-based morbidity surveillance since late 2005 in Asembo, in rural western Kenya and in Kibera, an informal settlement in Nairobi. The surveillance sites and design have been described previously [15], [16]. In brief, the population from July, 2006 included approximately 25,000 and 30,000 persons in the rural and urban areas, respectively [15], [16]. All participants must have resided permanently in the surveillance area for four calendar months [17]. In Asembo, malaria transmission is intense and occurs year-round [17]. In Kibera, the population lives in crowded conditions, with dirt paths between the dwellings and open sewers. Malaria is not endemic, but does occur particularly among those travelling to visit family in rural areas. Both sites have high adult prevalence of HIV at 15–17% (KEMRI/CDC unpublished data).

### Surveillance methods

Study participants receive free medical care for most acute conditions at a single referral health facility centrally-located in each site (Lwak Hospital in Asembo, Tabitha Clinic in Kibera). Patients are examined and diagnosed by clinical officers (similar to physician's assistants). Scannable paper questionnaires (TeleForm®, Cardiff™, California) or computerized databases (GFL Partners, Kenya) are completed on all sick visits, documenting symptoms, health-seeking, physical exam, diagnosis, treatment and outcome. Blood cultures are done on persons meeting one of three case definitions.

1. Severe acute respiratory illness (SARI), defined in persons ≥5 years old as cough or difficulty breathing and either temperature ≥38.0°C or oxygen saturation <90%, and in children <5 years old as those with clinical pneumonia as defined by WHO's Integrated Management of Childhood Illness [18].
2. Acute febrile illness, defined as a temperature ≥38.0°C, without SARI or other obvious source (e.g. bloody diarrhea), irrespective of malaria blood smear result (only the first two child and first two adult patients who meet this criteria per day are enrolled).
3. All patients, regardless of age, admitted for conditions unrelated to injury or obstetrics, at Lwak Hospital. (Tabitha Clinic does not have inpatient capacity.)

In addition, blood smears for malaria are done and read by KEMRI/CDC trained microscopists in the clinics, who undergo regular quality assurance evaluations. Stools are also collected, either in specimen cups or by rectal swabs, on patients with diarrheal illness (≥3 looser-than-normal stools in a 24-hour period), regardless of the presence of bloody diarrhea. Swabs of whole stool and rectal swabs were placed immediately in Cary-Blair transport medium and cooled to 4–8°C in insulated containers and shipped on the same day for immediate processing in KEMRI/CDC laboratories [19].

Community interviewers visit enrolled households every two weeks to inquire about illnesses. Symptoms are recorded and an abbreviated physical exam is done by trained field workers. Participants are asked if and where they sought health care. Using household visit data, we defined SARI as an episode with cough or difficulty breathing and documented fever and defined acute febrile illness as someone with reported fever without cough, difficulty breathing, or bloody diarrhea.

### Laboratory

Blood (7–10 ml for adults and 1–3 ml for children) was inoculated into commercially-produced broth bottles (BACTEC Plus™/F Aerobic Plus™ and Peds Plus™/F culture vials, Becton Dickinson, Belgium) and incubated in an automated BACTEC 9050 at 35°C for 1–5 days. Bottles with growth as indicated by an audible alarm were removed, Gram-stained and sub-cultured on standard media for bacteria identification using routine microbiologic techniques [20]. Standard methods were used to process stool specimens and identify pathogens [19]. *Salmonella* were identified by typical colony morphology and confirmed by biochemical typing and serotyping [20]. Antimicrobial susceptibility patterns were determined using standard Kirby-Bauer disc diffusion techniques [20].

*S.* Typhimurium isolates selected at random were lysed, digested with *Xba*l and *Bln*l restriction enzymes and DNA fragments separated by Pulse Field Gel Electrophoresis (PFGE) according to the PulseNet protocol [21]. The gels were stained with ethidium bromine, visualized and images were captured using gel documentation system and analyzed using BioNumerics software (Applied Maths, Inc, Austin, TX).

### Data analysis

Differences in proportions were assessed by chi-square tests and means by t-tests (Epi-Info™,version 3.4.3). Crude rates were calculated as the number of cases of NTS bacteremia per 100,000 person-years of observation (pyo). Each participating individual contributed person-time according to their dates of residence within the surveillance area from October 1, 2006–September 30, 2009 in Asembo and March 1, 2007–February 28, 2009 in Kibera.

Two adjustments were applied to crude rates. First, a multiplier was included for the percentage of persons visiting the referral clinics that met criteria for blood culture and did not have a culture done. The reasons for not getting cultured included the clinician not recognizing that the patient met the case definition, patient refusal, febrile patients presenting after two febrile persons had already been sampled that day, or an in-patient having already received intravenous antibiotics. The percentage of eligible patients who did not receive a blood culture was calculated separately for five age categories and for each indication for blood culturing. A second adjustment was made based on the percentage of persons identified with each of the indications for blood culture at the household visit who visited clinics other than Lwak or Tabitha for that illness. We assumed that those who visited another clinic for the same syndrome had a comparable severity and spectrum of etiologies to those that visited Lwak or Tabitha. 95% confidence intervals were calculated for crude rates using Fisher's method (PEPI, version 4.0×) and for adjusted rates using the delta method [22].

To estimate NTS bacteremia incidence in HIV-infected persons ≥18 years old in the rural site, we adjusted persons with unknown HIV status by applying the same proportion of HIV-positivity among patients with NTS bacteremia for whom HIV status was known. Population prevalence of HIV by age group was obtained from the home-based HIV testing initiative in the surveillance population in 2008 in which all adults were offered HIV testing (KEMRI/CDC unpublished data). Age-specific HIV prevalence rates were applied to obtain the age-specific person-years denominators. Similar adjustment factors were applied as described above.

### Ethical review

The protocol and written informed forms for surveillance and home-based HIV testing were reviewed and approved by the Ethical Review Board of KEMRI and the Institutional Review Board of CDC. For children less than 15 years old, parents, next of kin or guardians gave written informed consent to permit the children's participation in the study. Children aged 7–14 years also were required to give their written assent for participation.

## Results

Of 3,578 blood cultures done in the rural site, 155 (4.3%) had a pathogen isolated, of which 60 (38.7%) were NTS, the most common bacteria isolated, followed by *Streptococcus pneumoniae* (36.2% of isolates, Table 1). Twenty-three (38%) of rural NTS bacteremia patients were hospitalized. In the urban site, 2,138 blood cultures were done, with a pathogen isolated from 230 (10.8%), of which 7 (3.0%) were NTS; the most common bacteria isolated in the urban site were *Salmonella typhi* and *S. pneumoniae*. The median age of pediatric NTS patients in both sites was 2 years old and for patients ≥5 years was 32 years old. The sex distribution of NTS cases was comparable (rural–51% female; urban–57% female). Among rural children <5 years with positive blood cultures, 28/43 (65%) had NTS compared with 5/69 (7%) of urban children (Table 1, p<0.0001). In the rural area, NTS accounted for a greater proportion of bacteremia among febrile patients (72% of isolates) than among SARI patients (30% of isolates, p<0.005) or inpatients (36%, p = 0.008). In the urban area, the NTS isolation rate did not vary by indication for blood culture (SARI 2%, fever 5%, p = 0.3). Diarrhea was reported in 34% of patients with NTS bacteremia.

**Table 1:** Results of blood culture among patients with fever and/or severe acute respiratory illness, rural and urban Kenya, 2006–2009.

Age in years	0–4	5–9	10–17	18–49	>50	Total
**Rural Kenya**						
**Number patients (N)**	12,698	6,292	7,249	9,840	3,940	40,019
**Number of blood cultures, n (% patients)**	1,647(13.0)	708(11.3)	372 (5.1)	656 (6.7)	195(4.9)	3,578 (8.9)
**Positive blood cultures, n (%cultures)**	43(2.6)	13(1.8)	11 (3.0)	67(9.3)	18(9.2)	155 (4.3)
**NTS recovered, n (% positive cultures)**	28 (65.1)	6 (46.2)	1 (9.1)	19(28.4)	6(33.3)	60^a^ (38.7)
HIV positive/number tested* (%)	4/20 (20.0)	1/6 (16.7)	1/1(100)	9/11(81.8)	3/4 (75.0)	18/42 (42.9)
**Urban Kenya**						
**Number patients (N)**	18,315	5,314	2,744	11,185	775	38,333
**Number of blood cultures, n (% patients)**	1,024(5.6)	452 (8.5)	193(7.0)	447(4.0)	22(2.8)	2,138(5.6)
**Positive blood cultures, n (% cultures)**	69(6.7)	76 (16.8)	33(17.1)	51(11.4)	1(4.5)	230 (10.8)
**NTS recovered, n (% positive cultures)**	5(7.2)	1(1.3)	0 (0)	1(2.0)	0(0.0)	7 (3.0)^b^

a One patient, an HIV-positive individual, had a recurrence of NTS bacteremia infection after one month in the rural site.

b 0 of 1 (0%) adult with NTS was HIV-positive in Kibera.

The predominant serotype of *Salmonella* causing bacteremia differed in rural and urban Kenya, with NTS more common in the rural site and *Salmonella* Typhi more common in the urban site (Table 2) [23].

**Table 2:** NTS and *S.* Typhi cases by age category, rural and urban Kenya, 2006–2009.

Age category	Rural Kenya		Urban Kenya	
**<5 Years**	NTS Cases	28	NTS Cases	5
	Typhi Cases	1	Typhi Cases	35
	NTS: Typhi ratio	28.0	NTS: Typhi ratio	0.14
**>5 Years**	NTS Cases	32	NTS Cases	2
	Typhi Cases	12	Typhi Cases	100
	NTS: Typhi ratio	2.67	NTS: Typhi ratio	0.02

**Figure 1:**
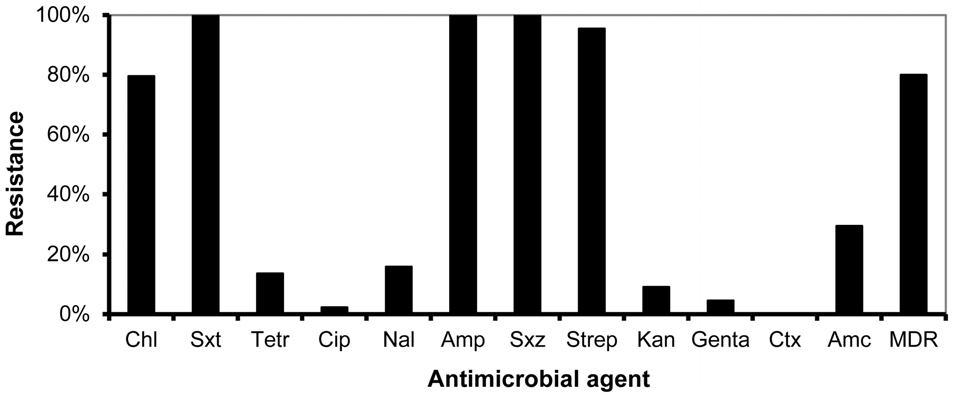
Antimicrobial resistance patterns among invasive S. Typhimurium (n = 45) in rural western Kenya, 2006–2009. Chl is chloramphenicol, sxt is trimethoprim-sulfamethoxazole, Tetr is tetracycline, Cip is ciprofloxacin, Nal is nalidixic acid, Amp is ampicillin, Sxz is sulfisoxazole, Strep is streptomycin, Kan is kanamycin, Genta is gentamycin, Ctx is ceftriazone, Amc is amoxicillin-clavulinic acid, Multi Drug Resistance (MDR) defined as resistance to chloramphenicol, trimethoprim-sulfamethoxazole and ampicillin.

**Figure 2:**
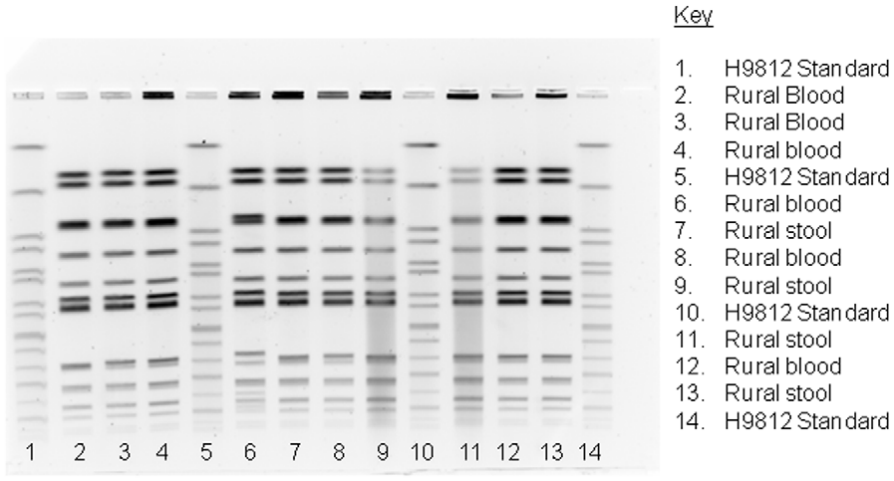
PFGE Gel patterns from XbaI restriction enzyme of Salmonella typhimurium isolates from blood and stool, western Kenya, 2006–2009.

There was a broader distribution of NTS serotypes in stool than blood (Table 3). *S.* Typhimurium was the predominant NTS blood culture isolate in both rural and urban Kenya accounting for 88% and 86% respectively. *S.* Typhimurium represented a greater percentage of stool isolates in the rural site (64%) than the urban site (21%, Table 3). Overall, 80% of *S.* Typhimurium isolates from blood were multi-drug resistant (MDR) in the rural site (Figure 1). The percentage of MDR among NTS blood isolates in the rural site did not change significantly during the course of the study – 2007, 11/13 (85%), 2008 9/10 (90%), 2009, 11/17 (65%). Of 14 *S.* Typhimurium blood isolates from the rural site that underwent PFGE, the predominant restriction pattern was found in 8 (57%) by Xba1 and 9 (64%) by Bln1 restriction enzymes, all of which were MDR (Figure 2). Three of six urban *S.* Typhimurium isolates were MDR, two of which had the same predominant PFGE pattern (by both Xba1 and Bln1) as the rural isolates.

**Table 3:** NTS stool and blood culture isolates, rural and urban Kenya, 2006–2009.

Category	Rural Kenya		Urban Kenya	
	(n = 60)		(n = 7)	
	Serotype	Number (%)	Serotype	Number (%)
**Blood Isolates**	Typhimurium	53 (88.3%)	Typhimurium	6 (85.7%)
	Enteritidis	6 (10.0%)	Enteritidis	1 (14.3%)
	Heidelberg	1 (1.7%)		

	(n = 33)		(n = 14)	
	Serotype	Number (%)	Serotype	Number (%)
**Stool Isolates**	Typhimurium	21 (63.6%)	Enteritidis	4 (28.6%)
	Enteritidis	3 (9.1%)	Typhimurium	3 (21.4%)
	Virchow	3 (9.1%)	Virchow	1 (7.1%)
	Heidelberg	2 (6.1%)	Heidelberg	1 (7.1%)
	Newport	2 (6.1%)	Muenchen	1 (7.1%)
	Aberdeen	1 (3.0%)	Agona	1 (7.1%)
	Chailey	1 (3.0%)	Stanleville	1 (7.1%)
			Haifa	1 (7.1%)
			Bovismorbificans	1 (7.1%)

**Figure 3:**
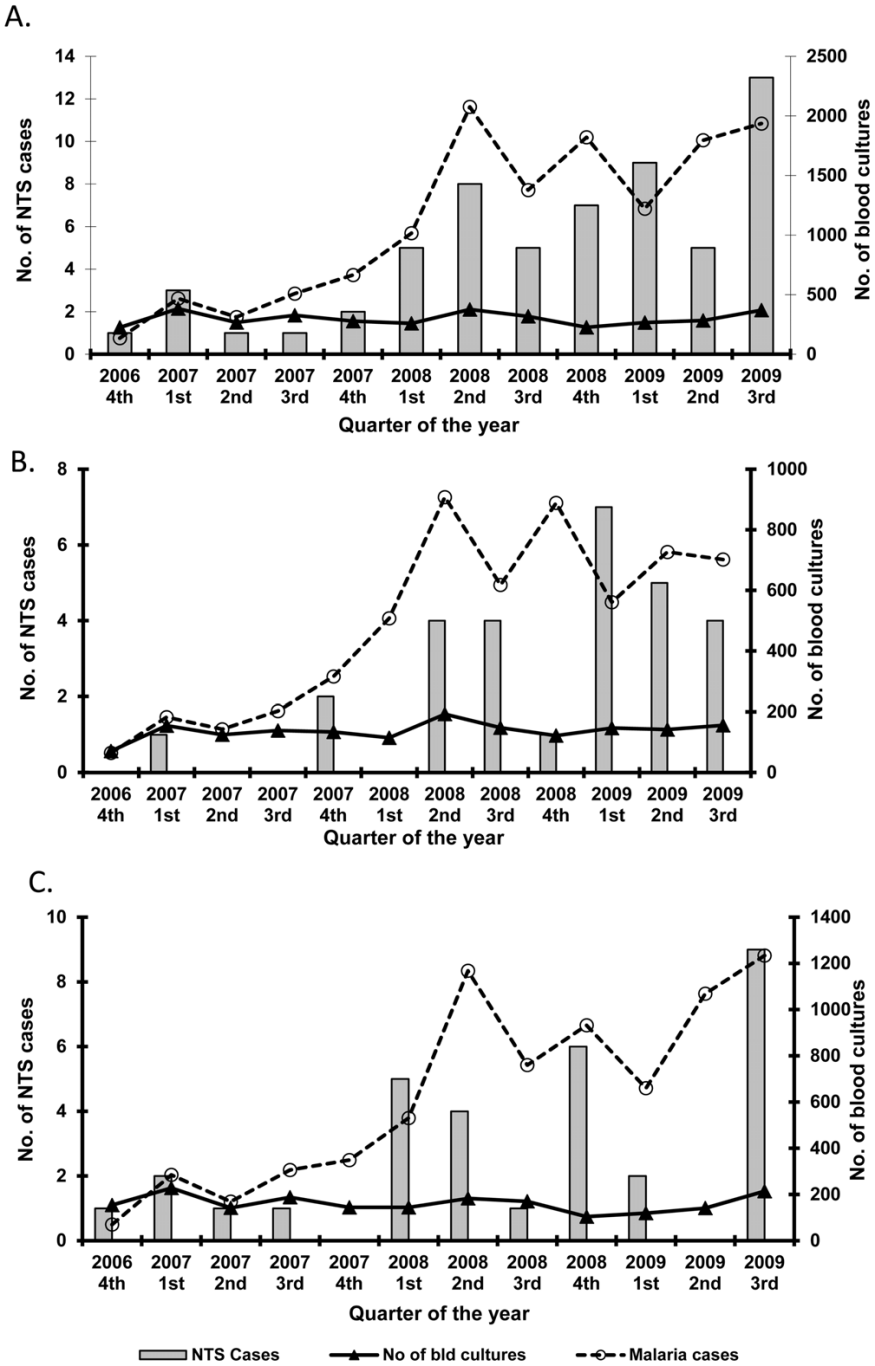
Numbers of NTS bacteremia, smear-positive malaria cases and blood cultures done by quarter of the year, rural western Kenya, 2006–2009. A. All persons (spearman rank correlation coefficient, 0.87, p = 0.0003). B. Children <5 years old (spearman rank correlation coefficient, 0.66, p = 0.018). C. Persons ≥5 years of age (spearman rank correlation coefficient, 0.43, p = 0.18).

There was no clear seasonal pattern to NTS bacteremia in either site. In the rural site, a concomitant increase in the number of NTS bacteremia cases and positive malaria cases was observed in 2008–9 (Figure 3, Spearman's correlation, p = 0.018 for children, p = 0.16 adults, p = 0.0001 overall). Positive malaria blood smears were more common among NTS bacteremia patients (20%) compared with patients who were blood culture positive for pathogens other than NTS (10%), although not statistically significant (p = 0.14). The number of blood cultures and isolation rate for bacteria other than NTS from blood culture did not change substantively during the surveillance period, suggesting the rising incidence of NTS bacteremia was not due to methodological changes. Similar temporal trends were not seen in Nairobi.

In the rural site, 18 (43%) of 42 patients with NTS bacteremia whose HIV status was known were HIV-positive (Table 1). The HIV prevalence among NTS bacteremic patients >18 years was 80%, which was substantially higher than the adult HIV prevalence in the community (17%, KEMRI/CDC unpublished data). The one adult patient for whom HIV status was known in the urban site was HIV-negative.

Overall crude and adjusted NTS incidence was higher in rural Kenya (78 per 100,000 pyo and 580 per 100,000 pyo, respectively) than in urban Kenya (13 per 100,000 pyo and 57 per 100,000 pyo, respectively) (Table 4, p<0.0001 for urban: rural difference for both crude and adjusted rates). In both sites, NTS incidence was highest in children <5 years. In the rural site, the crude rate among HIV-infected persons >18 years was 334 per 100,000 pyo (95% CI 274–394), with an adjusted rate of 1,555 per 100,000 pyo (95% CI 956–2,155).

**Table 4:** Crude and adjusted incidence rates of NTS bacteremia among rural and urban populations of Kenya, 2006–2009.

AgeIn years	Site	NTS(n)	Pyo*	Crude Rate per 100,000 Pyo(95% CI)	Rate Extrapolation 1** per 100,000 Pyo(95% CI)	Rate Extrapolation 2*** per 100,000 Pyo(95% CI)
**0–4**	Rural	28	13,572	206 (138–298)	759 (542–916)	2085 (1181–2990)
	Urban	5	9,595	52 (17–122)	208 (95–322)	260 (102–419)
**5–9**	Rural	6	11,312	53 (19–115)	150 (82–218)	389 (106–672)
	Urban	1	8,049	12 (0.12–92)	25 (5–44)	37 (1–73)
**10–17**	Rural	1	16,756	6.0 (0.18–33)	18 (0–43)	24 (0–62)
	Urban	0	8,017	0 (0–46)	0 (NA)	0 (NA)
**18–49**	Rural	19	25,015	76 (46–119)	155 (106–206)	367 (186–550)
	Urban	1	26,752	3.7 (0.15–27)	7.5 (0–18)	11.2 (0–31)
**>50**	Rural	6	10362	58 (21–126)	145 (86–204)	232 (112–351)
	Urban	0	2,120	0 (0–174)	0 (NA)	0 (NA)
**Overall**	Rural	**60**	**77,017**	**78 (59–100)**	**230 (142–317)**	**580 (229–934)**
	Urban	**7**	**54,535**	**13 (5–26)**	**44 (0–89)**	**57 (0–122)**

*Pyo = person-years of observation.

**Extrapolated for patients meeting indication for blood culture who were not cultured in the clinic.

***Extrapolated for patients meeting indication for blood culture who were not cultured in the clinic and those with same illness syndromes at the home visit who sought care at area clinics besides Lwak and Tabitha clinics.

Among the 7 NTS-associated deaths (2 children and 5 older persons), 1 occurred in the first 30 days after the date of the positive blood culture, 3 died from 31–60 days, and 3 died from 61–90 days afterwards. The 30-day case-fatality ratio (CFR) was 0% for children and 3.1% for older persons; the 90-day CFR was 7.1% for children and 15.6% for older persons. Compared with patients of the same age group who had a blood smear positive for malaria, but were not bacteremic, the 90 day mortality among NTS bacteremia cases in the rural Kenya site was 9 times higher for patients <5 years old and 68 times higher for patients ≥5 years old (Table 5). In contrast, the 90 day mortality among patients with NTS bacteremia was not significantly higher than that among patients with bacteremia due to other bacteria. Verbal autopsies on these 7 patients determined that 6 died of HIV/AIDS and 1 died of tuberculosis. No NTS-associated deaths occurred in the urban site.

**Table 5:** 90-day mortality among NTS bacteremia and malaria blood smear positive patients, rural^1^ Kenya, 2006–2009.

Age	NTS cases 90 day mortality rate (per 1,000 pyo)^2^	B/S Positive malaria cases 90 day mortality rate (per 1,000 pyo)	Risk Ratio for 90 day mortality NTS: Malaria (95% CI)	Non-NTS bacteremia 90 day mortality rate (per 1,000 pyo)	Risk Ratio for 90 day mortality NTS: Other pathogen (95% CI)
<5 Years	298	33	9.0 (2.4–37)	288	1.0 (0.14–7.9)
>5 Years	683	10	67 (26–172)	491	1.39 (0.49–4.0)

1 There were no NTS related deaths reported in the urban area during the study period.

2 90-day mortality rate calculated as deaths in the 90 days after positive blood culture or malaria blood smear, annualized to 1,000 person-years of observation.

## Discussion

NTS was the leading isolate from bloodstream infections in rural Kenya, with incidence in the higher range of those found in other rural African settings, where rates ranged from 88–330 cases per 100,000 pyo [1], [2], [4], [7], [9], [24]–[26]. In contrast to rural Kenya, NTS bacteremia rates were much lower in the urban informal settlement, where *Salmonella* Typhi predominated [23]. The most likely explanation for the rural-urban disparity in NTS bacteremia is that malaria is holo-endemic in the rural setting, while only occurring sporadically through importation in high altitude Nairobi (approximately 1,600 meters elevation). The association between malaria and NTS bacteremia is well-described [25], [27]–[32]. NTS bacteremia is higher in those with current or recent malaria and malarial anemia [2], [9], [26]–[30]. NTS rates were higher in the malaria season in Gambia [7] and decreased concomitantly with a decreasing trend in malaria prevalence over several years in Gambia, Tanzania and coastal Kenya [25], [31], [32]. The association between NTS bacteremia and malaria is likely multi-factorial, and may include hemolysis leading to dysfunctional macrophage phagocytosis and release of free iron promoting bacterial growth [2], [25], [27]. The risk of NTS bacteremia might be particularly high with malarial anemia, which is very common among children in western Kenya [2], [28]. The increasing rate of NTS bacteremia from 2008 onwards in western Kenya likely resulted, in part, from an increasing malaria prevalence in the area [33].

Besides malaria, there are several other reasons for the rural/urban difference in invasive *Salmonella* epidemiology. A possible reason for high rates of typhoid in the urban site could be related to the crowded living conditions and poor water and sanitation, conditions more similar to Asian mega-cities where typhoid predominates [23], [34]. The lower rates of NTS bacteremia in the urban site might also be related to the observation that those who are terminally ill tend to leave Nairobi to go back “up-country” to their ancestral homes where they would prefer to die. As NTS bacteremia occurs more frequently in end-stage AIDS, this migratory phenomenon would tend to decrease NTS bacteremia in the city and increase it in rural areas. Another possible explanation for the low urban NTS rates is that people living in cities have less exposure to risk factors for NTS. There is some evidence from developed countries that exposure to domestic animals, particularly chickens which are common in rural Kenya, might be a risk for NTS disease [6], [8], [27].

The high burden of NTS in adults in the rural site is also influenced by the high prevalence of HIV, as has been shown in other African settings [13], [27], [35]–[40]. The adjusted rate of NTS among HIV-infected adults we found of 1,555 per 100,000 pyo was slightly lower than that in Malawi (8,000 per 100,000), Uganda (1,900 per 100,000) and elsewhere in Kenya (1,600–7,500 per 100,000) [36], [38]–[40]. This lower rate in Kenya might be due to the institution of the home-based HIV counseling and testing program in 2008 that identified many HIV-infected persons in early stages of infection (median CD4 400 cells/mm^3^), when they would be less susceptible to NTS bacteremia, as well as leading to the initiation of cotrimoxazole prophylaxis among many HIV-infected persons, which has been shown to prevent NTS bacteremia [41]. Almost all deaths following NTS bacteremia in our study were HIV-related, according to verbal autopsy. This suggests that while NTS bacteremia might not be the proximate cause of death, it can serve as an indicator of end-stage AIDS and of imminent death from other HIV-related causes. Immune reconstitution with anti-retrovirals and/or cotrimoxazole prophylaxis can reduce the risk of NTS bacteremia [36], [38], [41]. Third, because we not only cultured febrile patients, but also those with severe acute respiratory illness, we might have identified more NTS bacteremia. NTS bacteremia commonly presents with respiratory symptoms in children, although its role in causing pneumonia based on lung aspiration studies is doubtful [2], [42]. Alternatively, patients with severe respiratory illness due to another cause can be simultaneously bacteremic with NTS, particularly among HIV-infected persons [2], [11], [13], [35].

We found a lower mortality from episodes of NTS bacteremia than other studies. Because free, high-quality care was offered to surveillance participants, ill persons likely came to the clinic at earlier stages of illness, so that milder cases of NTS bacteremia might have been captured, as suggested by the observation that 62% of NTS bacteremia patients in the rural site were treated as outpatients. Most studies have been done among hospitalized patients, where case-fatality rates for NTS bacteremia were much higher than we found, ranging from 4–27% for children and from 22–47% for adults [27].

*S.* Typhimurium was the dominant serotype causing bloodstream infections in both urban and rural sites, in contrast to a much broader serotype distribution among contemporaneous NTS stool isolates [2], [3], [6], [8]. Rises in multi-drug resistant *S.* Typhimurium have been temporally related to increased numbers of NTS bacteremia cases in Malawi from 2001–2004 [13]. Genomic sequencing of these invasive multi-drug resistant *S.* Typhimurium isolates from Malawi, and of contemporaneous isolates from coastal and urban Kenya, revealed a similar clone, ST313 [14]. It was postulated that genomic degradation in this NTS clone created characteristics more similar to *Salmonella* Typhi, changes that led to greater invasive potential [14]. Without genetic sequencing, we lack conclusive evidence that the predominant multi-drug resistant strain in our sites were the ST313 clone, although the predominant PFGE pattern had an antimicrobial resistance profile similar to previously identified ST313 isolates from Kenya [14].

Our study had several limitations. First, in making adjustments of bacteremia rates, we made certain assumptions – NTS isolation rates would be the same among those meeting indications for blood culture who did not get cultured and that those patients under surveillance who attended another clinic for their illness would also have similar isolation rates to those who went to Lwak and Tabitha. Both assumptions might be subject to biases. Nonetheless, the crude rates are clearly an underestimate of the true rate in the community, due to limited health-care utilization and insensitivity of blood cultures, and the adjustments provide reasonable upward adjustments, although should be considered the maximal rate. Second, only 60% of patients with NTS bacteremia had a confirmed HIV status and we assumed that those not tested had the same proportion HIV-positive as those tested. This assumption also might have been subject to differential bias. Third, NTS is a more robust and antibiotic resistant than the more fastidious Gram-positive bacteria, particularly *Streptococcus pneumoniae*, and therefore we can only claim that NTS was the most common isolate from blood culture in the rural site, rather than the most common cause of bacteremia.

Regardless of these limitations, NTS bacteremia clearly continues to cause a substantial burden of morbidity among children and HIV-infected adults in rural Kenya, warranting further investigation to define amenable risk factors, best treatment guidelines and new interventions, such as NTS vaccines currently under development [43].
